# Why is Skeletal Muscle Regeneration Impaired after Myonecrosis Induced by Viperid Snake Venoms?

**DOI:** 10.3390/toxins10050182

**Published:** 2018-05-01

**Authors:** José María Gutiérrez, Teresa Escalante, Rosario Hernández, Stefano Gastaldello, Patricia Saravia-Otten, Alexandra Rucavado

**Affiliations:** 1 Instituto Clodomiro Picado, Facultad de Microbiología, Universidad de Costa Rica, San José 11501-2060, Costa Rica; teresa.escalante@ucr.ac.cr (T.E.); alexandra.rucavado@ucr.ac.cr (A.R.); 2 Facultad de Ciencias Químicas y Farmacia, Universidad de San Carlos de Guatemala, Guatemala City 01012, Guatemala; rosariodamarish@gmail.com (R.H.); psaravia02@gmail.com (P.S.-O.); 3 Department of Physiology and Pharmacology, Karolinska Institutet, Stockholm SE-17177, Sweden; stefano.gastaldello@ki.se; 4 Precision Medicine Research Center, Binzhou Medical University, Laishan District, Guanhai Road 346, Yantai 264003, China

**Keywords:** snake venom, myonecrosis, envenoming, muscle regeneration, vasculature, extracellular matrix, snake venom metalloproteinases, phospholipases A_2_

## Abstract

Skeletal muscle regeneration after myonecrosis involves the activation, proliferation and fusion of myogenic cells, and a coordinated inflammatory response encompassing phagocytosis of necrotic cell debris, and the concerted synthesis of cytokines and growth factors. Myonecrosis often occurs in snakebite envenomings. In the case of venoms that cause myotoxicity without affecting the vasculature, such as those of many elapid snakes, regeneration proceeds successfully. In contrast, in envenomings by most viperid snakes, which affect the vasculature and extracellular matrix in addition to muscle fibers, regeneration is largely impaired and, therefore, the muscle mass is reduced and replaced by fibro-adipose tissue. This review discusses possible causes for such poor regenerative outcome including: (a) damage to muscle microvasculature, which causes tissue hypoxia and affects the inflammatory response and the timely removal of necrotic tissue; (b) damage to intramuscular nerves, which results in atrophy of regenerating fibers; (c) degradation of muscle cell basement membrane, compromising the spatial niche for proliferating myoblasts; (d) widespread degradation of the extracellular matrix; and (e) persistence of venom components in the damaged tissue, which may affect myogenic cells at critical points in the regenerative process. Understanding the causes of poor muscle regeneration may pave the way for the development of novel therapeutic interventions aimed at fostering the regenerative process in envenomed patients.

## 1. Introduction

Snakebite envenoming is a neglected tropical disease that kills and maims hundreds of thousands of people every year, especially in sub-Saharan Africa, Asia, Latin America and parts of Oceania [1,2]. The pathophysiology and clinical manifestations of these envenomings vary depending on the type of venom. Elapid snake venoms (family Elapidae) induce predominantly neurotoxic envenomings, resulting in neuromuscular paralysis that, if untreated, may lead to respiratory paralysis, whereas some elapid venoms also induce local necrosis and systemic myotoxicity [1,2]. In contrast, most viperid snake venoms (family Viperidae) cause local tissue damage (hemorrhage, necrosis, blistering, and edema) and systemic alterations associated with bleeding, coagulopathy, hemodynamic alterations and nephrotoxicity [1,2,3]. However, this basic pattern is an oversimplification of a highly complex and variable profile of clinical manifestations of envenomings, and specific pathophysiological profiles have been described for several snake venoms [4,5].

Acute muscle damage, i.e., myonecrosis, is a common manifestation of snakebite envenoming. In some cases, such as those inflicted by various Australian terrestrial elapids, sea snakes, and few viperid species, such as the South American rattlesnake, systemic myotoxicity, i.e., rhabdomyolysis, develops. This, in turn, may contribute to the acute kidney injury often associated with these envenomings [1,2,6,7]. On the other hand, envenomings by most viperid snakes are characterized by local pathological events, including local myonecrosis [1,2,5,8]. Moreover, viperid venoms cause prominent damage to other cells, blood vessels, and the extracellular matrix (ECM) components of muscle tissue, thus generating a pathological scenario associated with a deficient skeletal muscle regeneration [9]. Consequently, some envenomed patients end up with permanent tissue damage and sequelae, thus having a great impact in their quality of life and generating a wave of social suffering in families and communities alike [2]. Despite its relevance and impact, the basis of the poor muscle regeneration in viperid snakebite envenomings has not been completely understood. The present review summarizes the basic aspects of the process of skeletal muscle regeneration and discusses possible causes for the deficient regenerative outcome in snakebite patients, together with possible therapeutic interventions aimed at improving regeneration.

## 2. A Brief Outlook into the Pathogenesis of Myonecrosis Induced by Snake Venoms

Snake venoms are chemical cocktails that encompass many proteins classified within a limited number of families [10]. Venoms of species of the family Elapidae predominantly contain phospholipases A_2_ (PLA_2s_) and representatives of the three finger toxin family (3FTx), in addition to lower amounts of proteins from other families. In contrast, venoms from the family Viperidae are predominantly composed of various proportions of PLA_2_s, zinc-dependent metalloproteinases (SVMPs) and serine proteinases, in addition to variable amounts of C-type lectin-like proteins, disintegrins and other types of proteins [2,10].

Venom PLA_2_s are the most abundant myotoxic components in elapid and viperid snake venoms [11]. Catalytically-active PLA_2_s and PLA_2_ homologs devoid of enzymatic activity induce acute muscle damage by disrupting the integrity of skeletal muscle plasma membrane, i.e., the sarcolemma. Such disruption occurs through enzymatically-dependent and -independent mechanisms [11,12]. Plasma membrane damage is followed by a rapid influx of calcium ion from the extracellular fluid, following a steep gradient that occurs across this membrane. Consequently, a series of intracellular degenerative events ensue, such as myofibrillar hypercontraction, mitochondrial damage, and activation of calcium-dependent intracellular proteinases (calpains) and PLA_2_s, which rapidly bring the cell beyond the “point-of-no-return”, causing irreversible cell damage [11,12]. Besides PLA_2_s, other venom components contribute to acute muscle damage, such as the elapid cytotoxins (cardiotoxins) of the 3FTx family, which also act by perturbing plasma membrane integrity [13] and small myotoxins present in rattlesnake venoms [14].

In addition to myotoxic PLA_2_s, viperid venoms contain hemorrhagic SVMPs which contribute to the pathogenesis of muscle necrosis by generating ischemia in the tissue as a consequence of the profuse extravasation that occurs secondarily to the damage of capillary blood vessels by the action of venom metalloproteinases [15]. On the other hand, the increment in interstitial pressure within muscle compartments, as a consequence of the profuse extravasation of plasma caused by viperid venoms, may contribute to the impaired perfusion and ischemia in muscle tissue. Hence, in the case of viperid venoms that contain myotoxic, hemorrhagic and edema-forming toxins, myonecrosis occurs by a combination of the direct action of myotoxins on muscle fibers, as well as indirectly, through the ischemia resulting from venom-induced vascular alterations.

## 3. The Process of Skeletal Muscle Regeneration

Skeletal muscle tissue has the capacity to regenerate after a variety of pathological conditions [16]. Muscle regeneration in adult life recapitulates processes and programs of gene expression which occur during embryonic muscle development [17,18,19,20]. In normal muscle, a population of quiescent cells, satellite cells, is present at the periphery of muscle fibers outside the plasma membrane and within the basement membrane [21]. These cells have inconspicuous ultrastructural morphology and become activated after damage to muscle fibers. Upon activation, a percentage of satellite cells undergo a differentiation program, evolving to myoblasts which then fuse to form myotubes; these multinucleated cells then differentiate into adult muscle fibers [20]. In turn, a number of activated satellite cells return to a quiescent condition constituting the reserve population of myogenic cells [17,18]. The different stages in the activation and differentiation of satellite cells are marked by a variable expression of myogenic transcription factors. Quiescent satellite cells express Pax7, whereas activation results in the expression of MyoD, and myogenin is expressed later on in the differentiation process [17,19,20].

The process of muscle regeneration involves a highly orchestrated series of events that accompany the described activation and differentiation of myogenic cells. Furthermore, significant evidence suggests that, while satellite cells represent an important determinant for tissue regeneration, a “qualified” environment is necessary to achieve functional results [22]. To be successful, these processes occur concomitantly with a complex and dynamic interplay of local inflammatory phenomena and of remodeling of ECM. An acute inflammatory reaction is elicited after muscle damage, characterized by increments in vascular permeability and by an inflammatory infiltrate which, initially, is composed of neutrophils, and then by macrophages [23,24,25,26]. In addition to these infiltrating inflammatory cells, skeletal muscle has a rich population of resident macrophages, CD8+ cytotoxic T cells, regulatory T cells, and mast cells which comprise a complex inflammatory landscape which directly influences the regenerative processes [23,27,28]. In addition to removing necrotic and apoptotic debris in damaged muscle, some of these cells synthesize a variety of cytokines, such as interferon-γ (IFN-γ) and tumor necrosis factor-α (TNF-α) which influence the regenerative outcome [29,30]. The presence of immune signals and growth factors promote the up- and down-regulation of muscle specific genes that control the activation of satellite cells. The concerted release of these chemical signals is necessary to orchestrate an effective regenerative process [22].

Activated macrophages in damaged muscle initially belong to the M1 inflammatory macrophage phenotype and, later on, through the action of cytokines such as interleukin-10 (IL-10), M2 non-inflammatory macrophages predominate [23,31]. This switch is associated with the expression of genes that are characteristic of terminal differentiation of regenerating fibers. However, the scenario is more complex than that, since M1- and M2-type macrophages correspond to two extremes of a phenotypic spectrum encompassing variations in markers and functions, thus revealing a complexity that goes beyond this basic dichotomy [25,26,28]. Alterations in the removal of necrotic debris, and in the orderly appearance of inflammatory cells and mediators, including the shift from M1- to M2-type macrophages, preclude a successful regenerative response [23].

An interplay also occurs between myoblasts and myotubes, inflammatory cells and the population of resident cells of the fibro-adipogenic lineage which, upon activation, contribute to the synthesis and remodeling of ECM, with the involvement of matrix metalloproteinases (MMPs), the ADAMs (A Disintegrin and Metalloprotease) family of transmembrane glycoproteins, and plasmin and the associated plasminogen activation (PA) system, among others (reviewed in [32]). The regulated expression of MMP-2, MMP-9 and MT1-MMP is critical during skeletal muscle regeneration [33,34,35], although important roles for other members of the MMP family, such as MMP-13 and MMP-1, have been also proposed [36,37,38]. The balance between MMPs and their tissue inhibitors (TIMPs) regulates the turnover and remodeling of the ECM during physiological processes, such as angiogenesis and neovascularization [34,35,39], thus having a direct impact on tissue remodeling, including the deposition of collagens and fibronectin required for muscle regeneration [23,40,41].

Several growth factors and cytokines, secreted by myogenic and inflammatory cells, as well as those released from storage sites at the ECM, play a key role in myogenic cell proliferation and differentiation [17,18,42]. These include hepatocyte growth factor (HGF), fibroblast growth factor (FGF), transforming growth factor β (TGFβ), myostatin, insulin-like growth factor 1 (IGF-1), tumor necrosis factor β (TNF-β), tumor necrosis factor α (TNF-α), interferon γ (IFN-γ), interleukin 6 (IL-6), platelet-derived growth factor (PDGF), and leukemia inhibitor factor (LIF), among others [19,22,23].

**Figure 1:**
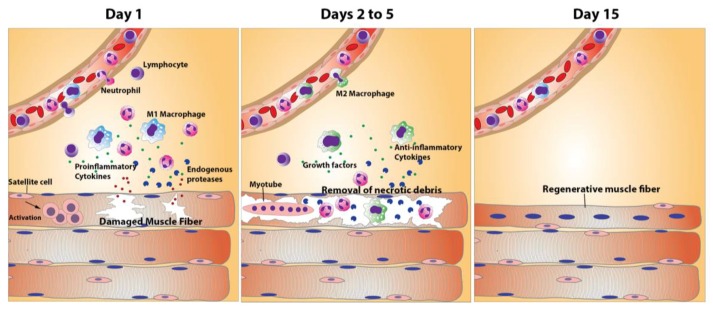
Basic scheme of normal skeletal muscle regeneration. Skeletal muscle necrosis, which can be induced by a variety of agents and clinical conditions, generates during the first day an inflammatory response characterized by activation of resident immune cells and by the influx of neutrophils and M1 pro-inflammatory macrophages. In this inflammatory milieu, there is synthesis of pro-inflammatory cytokines and endogenous proteinases. At the same time, myogenic satellite cells are activated and become myoblasts which then replicate. During Days 2–5, the inflammatory response evolves and results in the removal of necrotic muscle debris by inflammatory phagocytic cells; simultaneously, growth factors are synthesized by various types of cells. A shift in the phenotype of the predominant population of macrophages takes place, from pro-inflammatory M1 type to anti-inflammatory M2 type. Myoblasts fuse to form multinucleated myotubes. At Day 15, myotubes have become regenerated muscle fibers, characterized by centrally-located nuclei. Along this process, there is turnover of extracellular matrix with changes in its composition. In conditions where muscle vasculature and nerve supply are not affected, the regenerative process develops normally and successfully.

Basic requirements for a successful muscle regenerative response include an intact blood supply, the restitution of neuromuscular junctions and the presence of a structurally-intact basement membrane surrounding necrotic muscle fibers [18,43,44]. An intact vasculature ensures oxygen and nutrient supply, and contributes to a coordinated inflammatory response, with the transmigration and movement of leucocytes from the microvasculature to the affected tissue. Innervation is a requisite for full differentiation of regenerating fibers, whereas basement membrane is a scaffold that demarcates the cellular space where myoblasts replicate and then fuse to form myotubes. Alterations in blood supply, innervation or basement membrane integrity have a direct negative impact in the success of the regenerative process. Figure 1 summarizes the main stages in the process of normal skeletal muscle regeneration.

## 4. Skeletal Muscle Regeneration after Myonecrosis Induced by Snake Venoms at the Clinical Setting

Acute skeletal muscle degeneration, i.e., myonecrosis, occurs in envenomings following bites inflicted by snake species of the families Elapidae and Viperidae [1,2,5,45,46]. Some venoms, such as those of various Australian terrestrial elapids, sea snakes, and subspecies of the South American rattlesnake (*Crotalus durissus*), contain potent myotoxins, mostly PLA_2_s [11], which induce systemic myotoxicity. Rhabdomyolysis in these cases is characterized by high increments in serum myoglobin concentration and creatine kinase (CK) activity, as well as by hyperkalemia. Such pronounced myotoxicity often causes acute kidney injury, mostly due to the toxicity of myoglobin in renal tubules [6,7,47]. In these cases, myotoxicity occurs in the absence of local tissue pathology associated with damage to the vasculature, ECM, and nerves. Therefore, in these circumstances, muscle regeneration proceeds normally and successfully, and generally no musculo-skeletal sequelae are described in these patients.

**Figure 2:**
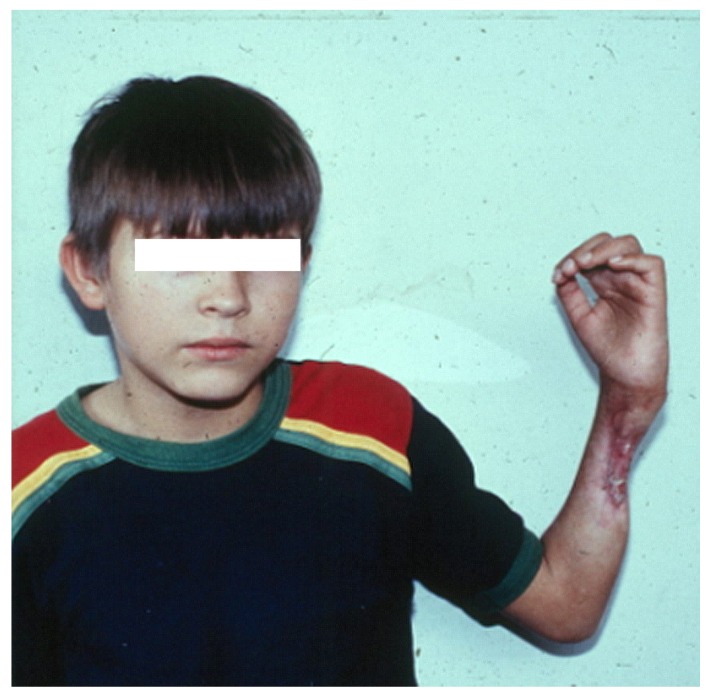
Patient suffering of permanent sequelae as a consequence of soft tissue necrosis secondary to a bite by a viperid snake (identified by his relatives as a “patoca”, i.e., *Porthidium* sp) in the Department of Antioquia, Colombia, while performing agricultural work. The patient was assisted by a local healer (“curandero”), without receiving antivenom, and only attended the hospital 30 days after the bite; the photo corresponds to the first day of admission. Drastic pathological changes occurred in muscle, tendons, nerves and bone, and the patient lost motor functions in his hand. Description of the case and photography courtesy of Dr. Rafael Otero-Patiño (Medellín, Colombia).

In contrast, envenomings by most viperid snakes cause, in addition to acute muscle necrosis, alterations to other components of the tissue where venom is injected, such as damage to the microvasculature resulting in hemorrhage, lesions to the arterial wall, thrombosis, nerve damage, and extensive ECM degradation [1,2,5,8,48]. Few elapid venoms, such as those of some cobra species (*Naja* sp.), also inflict local tissue damage, caused by the action of PLA_2_s and of cytotoxins (cardiotoxins), which are members of the 3FTx family characteristic of elapid venoms [13,49]. In envenomings in which muscle tissue damage involves various tissue components in addition to muscle fibers, the regenerative capacity of muscle is impaired, regeneration is deficient, and affected people are often left with permanent sequelae [2,50,51,52] (Figure 2). Thus, the different outcome, in terms of muscle regenerative capacity at the clinical level, in envenomings caused by purely myotoxic venoms and venoms that, besides muscle degeneration, affect other components in the muscle tissue, underscore the relevance of understanding the tissue context in which regeneration occurs and the key alterations that preclude a successful regeneration, an issue that has been explored to some extent at the experimental level.

## 5. Experimental Studies of Muscle Regeneration after Myonecrosis Induced by Snake Venoms and Toxins

### 5.1. Venoms and Toxins that Induce Myonecrosis without Affecting the Vascular Supply

The first experimental studies of muscle regeneration in tissue affected by snake venoms were performed by John Harris and colleagues, working with venoms and toxins of Australian elapids, mostly *Notechis scutatus* and *Oxyuranus scutellatus*, and their neurotoxic and myotoxic PLA_2_s notexin and taipoxin, respectively [53,54,55]. Using these and other myotoxic PLA_2_s, it was shown that a highly synchronous process of necrosis, inflammation and regeneration occurs [56]. Necrotic tissue is substituted by regenerative muscle, characterized histologically by fibers with centrally-located nuclei and by the presence of regenerative “split fibers” (“branched fibers”), with very limited fibrosis [57]. Similar observations were performed by other groups in models of muscle damage induced by elapid cardiotoxins [58,59,60]. As in the case of myotoxic PLA_2_s, cardiotoxins induce acute muscle damage by disrupting the integrity of skeletal muscle fiber plasma membrane without causing collateral damage to blood vessels and the basement membrane of muscle fibers [13,58]. Thus, venom myotoxic PLA_2_s and cardiotoxins have become useful tools for investigating various aspects of the process of muscle regeneration (see [56,59]).

Similar findings have been made with myotoxic PLA_2_s isolated from several viperid snake venoms, such as *Bothrops asper* [61,62], *B. jararacussu* [63], *B. nummifer* [64], *Agkistrodon contortrix* [65], and *Crotalus durissus terrificus* [66]. In these experimental studies of myonecrosis and regeneration in models in which there is no vascular damage, a similar process has been described: there is an initial phase, within the first 12 h, of acute muscle damage, secondary to the catalytically-dependent or -independent perturbation of muscle fiber plasma membrane integrity [11,12]. This is followed by an inflammatory response associated with edema and by a prominent leukocyte infiltrate [61,67]. Initially the predominant cells in the infiltrate are polymorphonuclear leukocytes, mostly neutrophils, which are then followed by a macrophage-rich infiltrate [67]. This inflammatory scenario is also characterized by the synthesis of a diverse set of mediators which can be identified in tissue homogenates or through the analysis of exudates collected from muscle tissue, including MMPs, cytokines, chemokines, growth factors, and others [68,69,70,71,72,73]. Activation of myogenic satellite cells occurs during these first phases of the reparative response, eventually becoming myoblasts that replicate in the space demarcated by the basement membrane of necrotic muscle fibers, and then fuse to form myotubes [55,56,61]. Satellite cells from neighboring, non-damaged muscle fibers are also activated, and migrate to the areas of degenerating muscle, probably contributing to the regenerative process [55,74].

**Figure 3:**
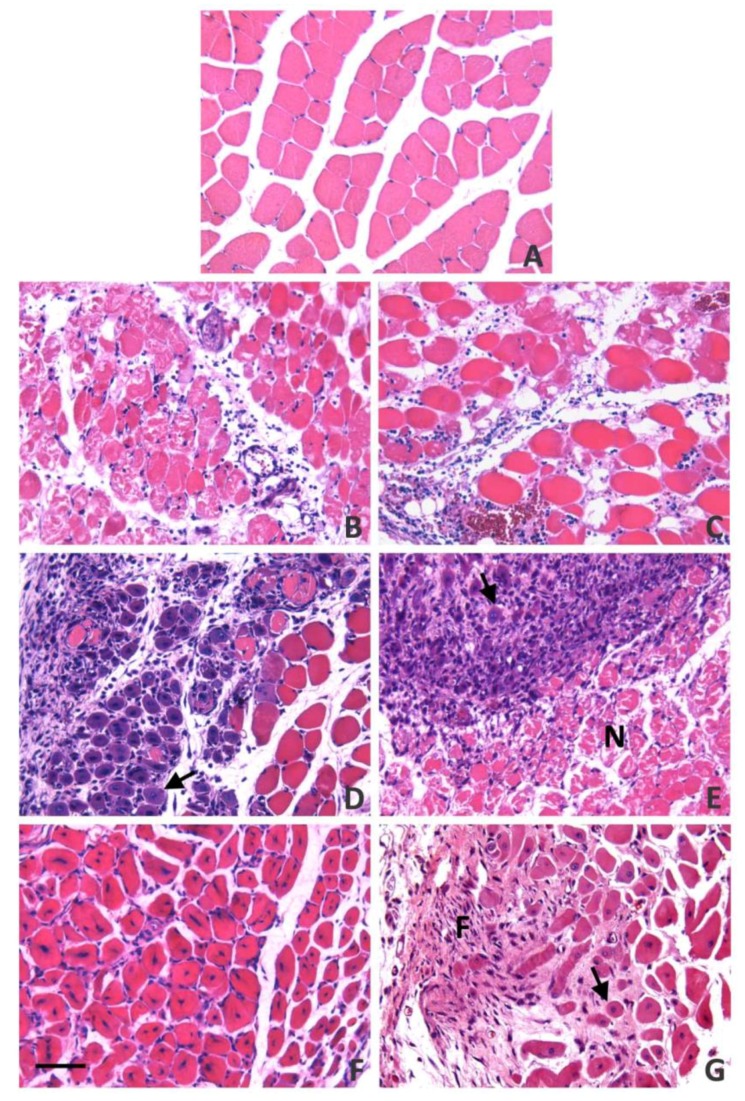
Light micrographs of sections of mouse gastrocnemius muscle at various time intervals after injection of: saline solution (**A**); a myotoxic phospholipase A_2_ purified from the venom of *Bothrops asper* (**B**,**D**,**F**); and crude venom of *Bothrops asper* (**C**,**E**,**G**). Muscle injected with saline solution shows a normal histological pattern. Muscle from mice injected with myotoxin shows widespread necrosis of muscle fibers and an inflammatory infiltrate 24 h after injection (**B**), whereas venom-injected tissue has, in addition to myonecrosis and infiltrate, areas of hemorrhage as a consequence of microvessel damage (**C**). By Day 4, there are abundant myotubes and immature regenerating muscle fibers with basophilic cytoplasm (arrow) in myotoxin-affected muscle (**D**), whereas, at the same time interval, muscle injected with venom presents areas with non-removed necrotic material (**N**) and others with inflammatory cells and few regenerating fibers of small diameter (arrow) (**E**). Fourteen days after injection of myotoxin, tissue shows a highly complete regeneration, with abundant regenerated fibers characterized by centrally-located nuclei and absence of fibrosis (**F**). In contrast, at this time interval, tissue from mice injected with venom (**G**) shows a dispersed population of regenerating fibers of various sizes (arrow), and a fibrotic area (**F**), thus revealing an incomplete process of regeneration. Hematoxylin-eosin staining. Bar represents 100 µm. Figure prepared from unpublished material.

By three to five days after the onset of necrosis, abundant myotubes and small myofibers are observed as a consequence of myoblast fusion and, by Day 7, most of the damaged muscle has been substituted by a homogeneous collection of small regenerating fibers characterized by centrally-located nuclei and basophilic cytoplasm, with very little fibrosis [55,61,62,63]. The size of regenerating muscle fibers increases along time, and by four weeks they reach the diameter of normal muscle fibers [54,63]. Quantitative estimations of regenerated muscle area and of muscle fiber diameter after myonecrosis induced by myotoxic PLA_2_s show a highly successful regeneration [61,62]. This is in contrast to the deficient regenerative process occurring in muscle tissue after myonecrosis induced by venoms that, in addition to affecting muscle fibers, also disrupt other components of the tissue (see next sections) (Figure 3).

Nevertheless, studies have revealed some differences between normal muscle and regenerated muscle in models of myonecrosis induced by snake venom PLA_2_s. There is a slight increment in collagen fibers in the interstitial tissue of myotoxin-affected regenerated muscle [62]. In addition, “split” muscle fibers (also described as “branched” fibers) and centrally-located nuclei are present in regenerating muscle [54,57,61]. Changes also occur in the types of muscle fibers. When a myotoxin from the venom of *Agkistrodon contortrix* was injected subcutaneously in the hind limb of mice, examination of soleus and gastrocnemius muscles revealed that regenerating muscle showed an increment in type I fibers and a drop in type II fibers, with an intermediate stage in the process in which type IIC fibers are abundant [65,75]. Some contractile properties of regenerating muscle after myonecrosis induced by the venom of *Notechis scutatus* were not fully recovered even 12 weeks after envenoming, indicating that the sole histological analysis of muscle does not necessarily predict functional recovery during regeneration [76].

Although it is clear that myotoxic PLA_2_s and cardiotoxins do not affect the vascular supply, i.e., microvasculature and arteries, some observations suggest that the intramuscular nerve supply is altered by the action of myotoxins, especially in cases in which myotoxic PLA_2_s are also neurotoxic by acting at the presynaptic terminal [56]. In a model of necrosis induced by a myotoxic venom and PLA_2_ [54], it was described that nerve terminals and terminal internodes were affected, but that there was a rapid restoration of functional innervation. In muscle affected by a cardiotoxin, the typical morphological features of neuromuscular junctions (“en plaque type”) were transformed to “en grappe type” [77]. After injection of a myotoxic PLA_2_ from the venom of *Bothrops asper*, the number of intramuscular nerves in the necrotic zone was reduced in the acute phase of damage, as judged by immunostaining of neurofilament protein. However, by Day 28, the nerve density had increased, hence reflecting a process of reinnervation [62]. Thus, the alterations induced by myotoxic PLA_2_s or cardiotoxins in intramuscular nerves are restored and do not seem to have a major impact in the process of muscle regeneration. It is likely that reinnervation and repair of nerve terminals occur timely during the process of regeneration. Consequently, the size of regenerating muscle fibers in these models is similar to the size of mature normal muscle fibers [61]. In summary, muscle regeneration is largely successful after myonecrosis induced by venoms and toxins which primarily affect skeletal muscle fibers without damaging the muscle vasculature and the ECM.

### 5.2. Venoms and Toxins that Affect Other Components of Skeletal Muscle in Addition to Causing Myonecrosis

Experimental studies with viperid snake venoms have shown that, in addition to acute skeletal muscle damage, they induce vascular and nerve alterations, as well as ECM degradation, within muscle tissue [62,78,79]; in this context, there is generally a poor muscle regenerative response [61,62,80,81,82,83]. Viperid snake venoms comprise, in addition to myotoxic PLA_2_s, other types of toxins which affect various components of skeletal muscle besides muscle fibers. These include SVMPs, serine proteinases, and hyaluronidases, among others [84]. SVMPs play a key role in the pathogenesis of local tissue damage in these envenomings, since they induce local hemorrhage [15,85], degradation of the ECM [86], blistering and dermonecrosis [87], myonecrosis [88,89,90], arterial necrosis [90], and damage to intramuscular nerves [62]. Hyaluronidases in the venom also contribute to extracellular matrix degradation [91]. Such widespread alterations in various components of the muscular tissue have a significant impact in the regenerative process, as discussed in the next sections.

## 6. The Key Role of Vascular Damage and Its Implications in the Inflammatory Response and Regeneration

A consistent observation in muscle injected with viperid venoms, or with hemorrhagic SVMPs, is a rapid and prominent disruption of the microvascular network. SVMPs degrade key components of the capillary basement membrane, thus weakening the mechanical properties of the vessel wall, leading to capillary disruption due to the action of biophysical hemodynamic forces operating in the circulation [85,92]. This has been demonstrated quantitatively as a drop in capillary density and capillary/muscle cell ratio in histological preparations [61,93], as well as through histochemistry [78] and immunohistochemistry [62]. Likewise, fragments of basement membrane proteins are detected by Western blot and in proteomic analyses of exudates collected in the vicinity of muscle tissue injected with *B. asper* venom and a hemorrhagic SVMP [72,94,95]. Moreover, necrosis of smooth muscle in the wall of intramuscular arteries has been described after injection of viperid venoms or hemorrhagic SVMPs [48,78,81,90,96]. Such drastic alterations in the muscle vascular network are likely to contribute to the deficient regenerative process, since an adequate blood supply is a requisite for successful muscle regeneration [32,97]. Hypoxia impairs the formation and growth of regenerating muscle fibers and increases the loss of muscle mass during the first week after acute muscle damage [98].

**Figure 4:**
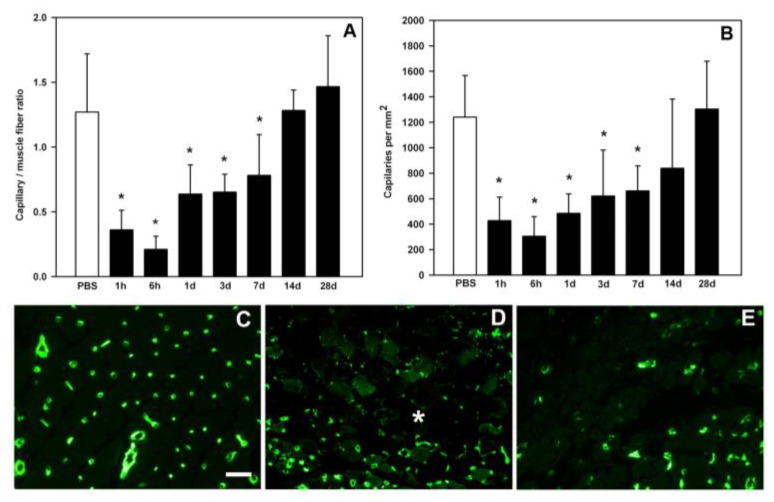
Damage to capillary network and revascularization in skeletal muscle injected with *Bothrops asper* venom, expressed as capillary/muscle fiber ratio (**A**) and capillaries per mm^2^ (**B**), assessed by immunohistochemistry with anti-CD31 antibody, a marker of endothelial cells. A drastic drop in capillaries occurred in the first hours after envenoming, followed by a process of revascularization. (**C**–**E**) Immunohistochemical stainings of endothelial cells in muscle tissue sections: 24 h after injection of saline solution (**C**); 24 h after injection of *B. asper* venom (**D**); and seven days after injection of venom (**E**). A reduction in the density of capillaries is observed in envenomed muscle after 24 h (asterisk) and seven days. Immunostaining of endothelial cells was performed with a rat anti-mouse CD31 monoclonal antibody, followed by polyclonal biotynilated rabbit anti-rat IgG and Streptavidin Alexa fluor 488. Bar represents 50 µm. Reproduced by [62], copyright 2011, PLOS ONE.

The issue of revascularization, i.e., the reconstitution of the capillary network after acute damage induced by hemorrhagic SVMPs and viperid venoms, is relevant to understand the regenerative process, and may shed light on the possible use of angiogenic factors as adjunct therapies to improve regeneration in these envenomings. Two studies have assessed revascularization in mouse experimental models. Santo Neto and Marques [93], working with the venom of *B. jararacussu* and using histological and ultrastructural methods, observed a poor revascularization in muscle, which took place at later time intervals and was incomplete. In contrast, Hernández et al. [62], using the venom of *B. asper* and quantifying capillaries by immunostaining of endothelial cells with anti-CD31 antibodies, observed a revascularization process that was underway by seven days and reached normal values of capillary density, as compared to control muscle tissue, by 14 days (Figure 4). The differences between these studies could be due to variations in the action of these venoms or to the different methods used to quantify capillaries. Regardless of this, both studies revealed that the capillary network was drastically affected by the action of these venoms, and that revascularization was incomplete during the first week after myonecrosis. Since critical events take place in the regenerative process during the first days after injury, the paucity of microvessels at this early time is likely to be a significant cause of poor regeneration in muscle affected by viperid venoms.

Such disruption in the microvascular network, added to the described alterations in larger vessels, may impact in the muscle regenerative process in, at least, two ways: (a) by generating ischemia and hypoxia; and (b) by affecting the orderly arrival and activation of inflammatory cells. Since regenerating muscle cells require an oxygen supply, the damage to vasculature, especially microvessels, is likely to affect the normal process of myogenesis owing to hypoxia. There is experimental evidence that myogenic cells show signs of degeneration during the first week after myonecrosis induced by hemorrhagic viperid venoms. The presence of “degenerating regenerative fibers”, i.e., small regenerating fibers with centrally-located nuclei showing signs of degeneration, in areas of fibrosis and scarce capillaries, has been described [78]. In another study, when comparing alterations in muscle injected with a myotoxic PLA_2_ and crude *B. asper* venom, no differences were observed in Pax-7 positive cells three days after muscle damage, suggesting a similar pattern of activation of satellite cells/myoblasts. However, TUNEL staining revealed that, in muscle injected with venom, there were areas with abundant TUNEL-positive regenerating cells, in contrast to tissue injected with the myotoxin [62], hence suggesting a regional increase in apoptotic regenerative fibers in envenomed muscle. Although preliminary, these findings may imply that satellite cells are activated and become myoblasts after viperid venom-induced muscle necrosis; however, the normal regenerative processes are somehow affected in this context, and regenerating cells degenerate. It can be speculated that appropriate signals required for regeneration are absent or limited within these first days after necrosis in conditions in which the microvasculature is affected, with a possible shift in the tissue reparative program from muscle regeneration to fibro-adipose proliferation [61,62,78,80].

The second mechanism through which vascular damage is likely to hamper muscle regeneration has to do with its impact on the inflammatory process required for an effective regeneration. Resident immune cells, i.e., resident macrophages, CD8^+^ T lymphocytes, and T_reg_ lymphocytes, are known to play a role in the activation and differentiation of muscle precursor cells [23]. Moreover, the inflammatory infiltrate reaching muscle tissue after necrosis, with an initial wave of neutrophils followed by macrophages, also contributes to the regenerative milieu by removing necrotic debris and by releasing cytokines, growth factors and other mediators which contribute to an orchestrated regeneration. A shift in the phenotype of macrophages, from pro-inflammatory M1 type to anti-inflammatory M2 type, is critical for the evolution of myogenic cells from a predominant landscape of myoblast proliferation to one of myoblast fusion and myotube formation [23]. It has been shown that alterations in this concerted interplay between myogenic and immune/inflammatory cells preclude an effective regeneration. In muscle affected by viperid snake venoms, tissue areas where the microvascular network has been severely affected by hemorrhagic SVMPs are characterized by a limited, or even absent, inflammatory infiltrate [61,80]. In these tissue areas, non-removed necrotic material is present even seven days after venom injection, whereas in models in which microvessels are not damaged, i.e., after myonecrosis induced by PLA_2_s, removal of necrotic material largely occurs within 24–48 h, and is followed by a normal regenerative process [54,61,63]. Necrotic fibers in which removal of cellular debris does not occur are characterized by the absence of proliferating myogenic cells within the basement membrane of necrotic fibers. In an experimental model of mice rendered neutropenic by the administration of a monoclonal antibody, the normal regenerative process characteristic of tissue affected by myotoxic PLA_2_s was impaired, and necrotic material within damaged cells was not cleared [99]. Furthermore, in this model, the number of macrophages was also reduced, thus implying that neutrophils release chemotactic mediators for the recruitment of inflammatory macrophages [23,99].

The paucity of inflammatory cells is likely to affect muscle regeneration in yet another way: infiltrating neutrophils, and especially M1-type macrophages, release mediators in the tissue, particularly cytokines and growth factors, which are critical for regeneration. Moreover, the transition in the predominant population of macrophages, from M1 to M2 phenotypes, shifts the scenario from a predominantly inflammatory phenotype to one characterized by promotion of myogenic cell replication and fusion, leading to the formation of abundant myotubes [23]. Thus, limiting the arrival of macrophages to the tissue jeopardizes the concerted process of regeneration. This paucity in macrophages in some areas of affected muscle may also alter the subtle equilibrium between proliferating myogenic cells and muscle resident fibro-adipogenic progenitor cells [23]. As a result, myogenic cells do not fulfill their program and, instead, there is proliferation of fibroblasts and pre-adipocytes, with the formation of a fibro-adipose tissue, as has been described in muscle injected with venoms that contain myotoxins and vascular-damaging toxins [61,78,80,100]. Under such suboptimal tissue milieu, there is also a modulation in the differentiation of myogenic cells, thus becoming fibroblasts or adipocytes [101,102].

## 7. The Complex Landscape of ECM Alterations in Viperid Venom-Induced Myonecrosis

A largely unexplored issue in this subject has to do with the role of viperid venom-induced ECM degradation in the deficient regenerative response. Upon muscle necrosis in several pathologies, ECM undergoes a finely regulated turnover process in which some components are degraded by the action of MMPs and other endogenous proteinases, whereas other proteins are synthesized, thus contributing to an optimal environment for myoblast proliferation and differentiation [32]. Viperid venoms contain high amounts of SVMPs and serine proteinases which can degrade ECM components. The action of venom hyaluronidase adds to the alterations of ECM [91]. In parallel, the onset of acute inflammation in necrotic muscle results in the overexpression of MMPs and other endogenous inflammatory proteinases [68,73] which also contribute to ECM turnover.

Proteomic studies on exudates collected from tissues injected with the crude venom of *B. asper* and with isolated SVMPs allowed the identification of fragments of many ECM proteins, including various fibrillar and non-fibrillar collagens, fibronectin, laminin, nidogen, tetranectin, vitronectin, lumican, decorin, tenascin, thrompospondin, fibrilin, chondroitin sulfate proteoglycan, and aggrecan [72,94,103], thus reflecting a widespread degradation of the matrix. ECM protein fragments were also detected in exudates from tissue injected with purified myotoxic PLA_2_s, probably due to the action of endogenous proteinases; however, their amounts were higher in samples from tissue injected with a SVMP [94]. It is therefore necessary to assess the possible effect of the hydrolysis of various ECM proteins on regeneration. Some of these fragments, in turn, may modulate the responses of inflammatory cells and of myogenic cells. Moreover, the preservation, after necrosis, of the structural scaffold of the basement membrane that surrounds skeletal muscle cells is a requisite for a proper regeneration, as this ECM structure delineates the space inside which myoblasts proliferate and fuse to form myotubes. Our unpublished immunohistochemical observations indicate that there is a partial loss of immunostaining of proteins in the basement membrane of muscle cells upon injection of hemorrhagic SVMPs. Thus, SVMP-induced degradation of muscle basement membrane and other components of the skeletal muscle ECM, and its effects on the regenerative process, is an issue that needs to be further investigated.

## 8. Damage to Intramuscular Nerves

An adequate nerve supply is another key requirement for skeletal muscle regeneration after injury [44]. Viperid snake venoms cause acute damage to intramuscular nerves. *B. jararacussu* venom induced widespread and progressive axonal damage in intramuscular nerve bundles in mice, together with myelin breakdown [79]. When nerve integrity was assessed by immunostaining with anti-S100 protein, the venom of *B. asper* caused massive intramuscular nerve pathology associated with axon loss and damage to the perineural structure [78]. Such extensive nerve damage might explain the observation that regenerating muscle fibers in tissue injected with *Bothrops* sp. venoms show an abnormally small diameter several weeks after envenoming, suggesting the presence of atrophic denervated fibers [61,78,80].

The effect of isolated venom components, i.e., myotoxic PLA_2_ and hemorrhagic SVMP, on intramuscular nerves was assessed by immunostaining with anti-neurofilament protein [62]. Drastic nerve damage was observed within the first days after injection of venom or toxins, as revealed by a reduction in the number of nerves per square millimeter. Thus, both PLA_2_s and SVMPs can affect nerve integrity. By Day 28, the number of nerves per area in the tissue of mice receiving venom, myotoxic PLA_2_ or hemorrhagic SVMP was similar to control muscle injected with saline solution, hence evidencing a re-innervation process. Nevertheless, when the number of axons per nerve area was quantified, there was a higher number in nerves of muscle treated with the PLA_2_, as compared to muscle injected with crude venom or SVMP [62]. These findings underscore a better re-innervation process after myonecrosis induced by myotoxins than when venom or SVMPs are injected, in agreement with the higher extent of regeneration in myotoxin-affected muscle. The basis for such deficient re-innervation after injection of venom or SVMP may have to do with the effects of microvascular damage and reduced tissue perfusion on nerves, or with degradation of the nerve basement membrane, which is determinant for nerve integrity [44], two issues that require further studies.

## 9. Does Residual Venom in the Necrotic Tissue Contribute to the Impairment of Regeneration?

Adding to this complex tissue pathology, the role of viperid venom components remaining in the affected muscle tissue has been explored as a possible cause for the poor regenerative response. After intramuscular injection of *B. asper* venom, the amount of venom components remaining in the necrotic tissue drops, and only a small percentage of the injected venom remains after several days [73]. Nevertheless, when homogenates from muscle collected five days after administration of *B. asper* venom were incubated with myoblasts in culture, there was a drop in the number of replicating myoblasts and a complete abrogation of myotube formation [73]. Noteworthy, these effects were completely abrogated when homogenates were previously incubated with antivenom antibodies, thus implying that traces of venom toxins present in the tissue can inhibit key steps in the process of muscle regeneration.

**Figure 5:**
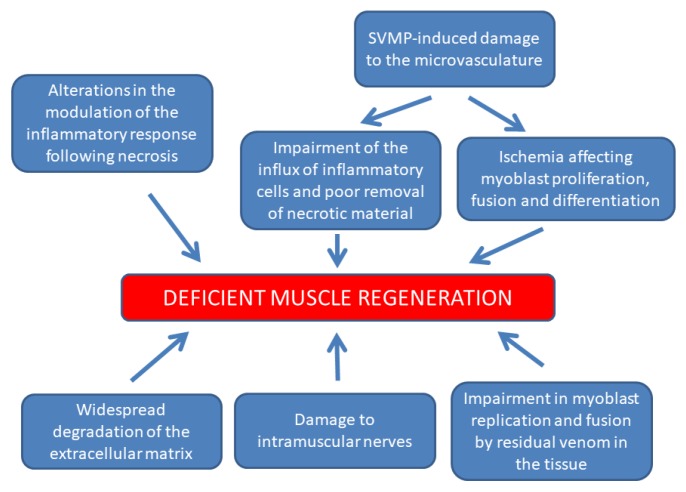
Summary of the main hypothetical factors that determine the poor outcome in skeletal muscle regeneration after myonecrosis induced by viperid venoms. In addition to myotoxicity, these venoms also induce damage to the microvasculature, the extracellular matrix and intramuscular nerves, hence causing tissue ischemia and alterations in the inflammatory response, including a reduction and delay in the arrival of phagocytic cells. The action of venom components remaining in the tissue when critical steps in the regenerative process are taking place are likely to contribute to the poor regenerative response.

Taken together, the experimental observations in muscle injected with viperid snake venoms indicate that critical steps in muscle regeneration occurring during the first days after the onset of myonecrosis are affected or modulated, impairing a successful regenerative process. These might be related to changes in the balance of inflammatory mediators and growth factors, variations in the timely influx of inflammatory cells, ischemic conditions secondary to vascular damage that impact on the differentiation of myoblasts, alterations in clues provided by the ECM, damage to intramuscular nerves, and deleterious actions on regenerating cells of venom components remaining in the tissue (Figure 5). Understanding which factors play the main role in the impairment of early regenerative events would be a critical step to devise novel therapeutic alternatives for people suffering snakebite envenomings.

## 10. Exploring Therapeutic Options to Improve Muscle Regeneration in Viperid Snakebite Envenoming

Although incomplete, our understanding of the possible factors that preclude an adequate regenerative response in skeletal muscle after necrosis induced by viperid venoms sheds light on possible therapeutic avenues to improve regeneration. Thus, by modulating factors and processes involved in regeneration, it should be possible to foster a more successful regenerative process. Since key decisions in the regenerating tissue occur during the first days after acute muscle damage, interventions directed at processes taking place during this window of time are likely to offer the best possibilities.

One approach involves the modulation of the inflammatory response. Since the kinetics of the inflammatory infiltrate play a key role in the removal of necrotic tissue and the activation of myoblast proliferation and fusion, understanding the mediators involved in chemotaxis and demise of these inflammatory cell populations may pave the way for the modulation of these processes [23]. It has been shown that increasing the local concentration of nitric oxide (NO) in affected muscle reduces inflammation and enhances muscle regeneration in muscle injected with a purified myotoxin [104] and with the venom of *B. jararacussu* [105]. However, administration of the anti-inflammatory drug deflazacort was detrimental for regeneration in muscle affected by the same venom [106], indicating that, depending on the inflammatory pathway being manipulated, the effect on regeneration varies. On the other hand, radicicol, an inducer of heat shock proteins, stimulated regeneration in muscle injected with the myotoxic PLA_2_ crotoxin [66]. Injection of macrophages of a variable phenotype, i.e., M1 or M2, at different times after muscle damage represents another possible approach to improve regeneration [107]. A key aspect in these experimental interventions is that, depending on the time of administration of drugs or cells, the effects on regeneration vary, hence underscoring the relevance of identifying the precise window of time when these interventions may be effective.

Hormones are also known to affect muscle regenerative capacity. Accordingly, administration of nandrolone, an anabolic androgenic steroid, increased the number of large regenerating fibers and the expression of MyoD-positive cells after necrosis by the venom of *B. jararacussu* [108], in agreement with the improvement of regeneration in animals treated with testosterone [109].

An alternative approach is the application of ultrasound to affected muscle. When this intervention was applied to muscle damaged by myotoxins or crude venoms, an improved regenerative response was observed. In the case of muscle damaged by cardiotoxin, ultrasonic treatment resulted in an increase in the cross-sectional area of myofibers as well as in myogenin RNA [110]. Likewise, structural and functional improvement was described in regenerating muscle after myonecrosis induced by *B. jararacussu* venom when ultrasound was applied [111]. On the other hand, gallium arsenide laser irradiation of muscle injected with the neuro/myotoxin crotoxin and with *Bothrops moojeni* venom also resulted in an improved regenerative response [112,113]. Further experimental studies with other snake venoms should be performed to accumulate evidence for the eventual testing of these therapies in snakebite cases at the experimental and clinical settings.

Based on the knowledge gained on the factors which are likely to determine the deficient muscle regeneration after injection of viperid venoms, several therapeutic interventions can be envisaged. These include, among others: (a) inhibiting the action of TGF-β, which favors fibrosis instead of myogenesis; (b) modulating the inflammatory components that promote or inhibit regeneration; (c) modulating the turnover of ECM by using metalloproteinase inhibitors or other agents; (d) promoting angiogenesis and revascularization; (e) administering ECM components which may induce a positive modulation of regeneration; (f) providing growth factors or platelet-rich plasma; and (g) injecting muscle-precursor cells, or inflammatory cells, previously stimulated in vitro. Table 1 summarizes examples of agents that might be tested along these lines.

The complexity and multifactorial nature of the muscle regeneration process suggests, however, that single interventions are unlikely to yield positive results. Instead, the simultaneous modulation of various factors involved in regeneration is probably needed. Thus, the design of experimental settings aimed at finding therapeutic options to improve muscle regeneration in viperid envenomings necessarily demands the interplay of agents and time intervals when these interventions are applied, thus making such elaborated designs a challenging task.

## 11. Concluding Remarks

Most viperid snake venoms induce drastic pathological alterations in muscle tissue. In addition to necrosis of muscle fibers, venoms cause microvascular damage leading to hemorrhage, widespread ECM degradation, and lesions to intramuscular nerves. In the context of this pathological scenario, the process of skeletal muscle regeneration is deficient and, consequently, there is tissue loss and substitution of muscle by fibrotic and adipose tissues. This translates into permanent sequelae which impact the quality of life of envenomed people and causes much suffering to families and communities. In contrast, venoms that induce muscle damage without affecting the vascular supply, such as those of many elapid and few viperid species, cause local and, sometimes, systemic myotoxicity, but the regenerative process develops adequately and the damaged muscle recovers.

Experimental evidence suggests that the causes of poor regeneration after viperid venom-induced myonecrosis are likely to depend on the alterations in muscle vasculature, ECM and nerve supply. The disruption of the microvascular network in muscle, as a consequence of the action of hemorrhagic SVMPs, generates an ischemic milieu which impairs the activation and differentiation of muscle precursor cells. Likewise, microvascular alterations preclude the orderly arrival of inflammatory cells which play a key role in conditioning the regenerative landscape by removing necrotic debris and releasing mediators that modulate inflammation and muscle differentiation. Degradation of ECM affects vital clues that orchestrate some aspects of the regenerative response, and disrupts the scaffolding role of the basement membrane of damaged muscle fibers. Finally, damage to intramuscular nerves precludes the timely reinnervation of regenerating fibers. An adequate understanding of this complex and dynamic tissue scenario demands integrative efforts, ideally under a systems biology perspective, which will provide a multicomponent view of the events taking place and the interactions between them [9].

Knowing the mechanisms involved in this poor regenerative response, and their interplay, is necessary to devise knowledge-based therapeutic interventions for improving muscle regeneration in people suffering viperid snakebite envenomings. Since antivenoms are only partially effective in the neutralization of venom-induced local tissue damage [131], especially when administered later on in the course of envenomings, an alternative approach for this clinical problem could be based on the improvement of muscle regeneration, a task that demands renewed interdisciplinary efforts.

## Figures and Tables

**Table 1:** Possible therapeutic interventions that could improve skeletal muscle regeneration after myonecrosis induced by viperid snake venoms.

Agents	Selected Actions for Improving Regeneration	References
Suramin	Antagonist of transforming growth factor-β (TGF-β). Stimulates myoblast differentiation and inhibits myostatin expression.	[114,115]
Minocycline	Protects against ischemia-reperfusion injury. Exerts anti-inflammatory effects.	[116,117,118]
Fucoidan	Promotes revascularization after ischemia. Regulates myogenic differentiation in vitro.	[119,120]
NaHS	H_2_S donor. Has pro-angiogenic properties after ischemia. Increases the expression of angiogenic factors.	[121,122]
Losartan	Inhibits TGF-β1 and reduces deposition of fibrotic tissue.	[123,124]
Laminin 111	Increases the expression of α7β1 integrin-type laminin receptor. Systemic administration in merosin-deficient congenital muscular dystrophy type 1A (MDC1A) prevents muscle pathology.	[125,126]
Stem cells/myoblast transplant	Repair of damaged skeletal muscle fibers by directly differentiating into myofibers and secretion of paracrine factors that promote tissue repair.	[127,128]
Platelet-rich plasma	Combination therapy with an anti-fibrotic agent improves skeletal muscle healing.	[129,130]
